# Prognostic Utility of Circulating Growth Factors in Aortic Valve Stenosis: A Pilot Study

**DOI:** 10.3390/medicina57010078

**Published:** 2021-01-18

**Authors:** Juris Hofmanis, Peteris Tretjakovs, Simons Svirskis, Gita Gersone, Dace Hofmane, Ulla Rozenberga, Leons Blumfelds, Guntis Bahs, Aivars Lejnieks, Vitolds Mackevics

**Affiliations:** Faculty of Medicine, Riga Stradins University, 16 Dzirciema Str., LV-1007 Riga, Latvia; jhof.cor@gmail.com (J.H.); simons.svirskis@rsu.lv (S.S.); Gita.Gersone@rsu.lv (G.G.); dace.kardio@gmail.com (D.H.); ulla.rozenberga@gmail.com (U.R.); Leons.Blumfelds@rsu.lv (L.B.); Guntis.Bahs@rsu.lv (G.B.); Aivars.Lejnieks@rsu.lv (A.L.); Vitolds.Mackevics@rsu.lv (V.M.)

**Keywords:** aortic valve stenosis, growth differentiation factor 15, angiopoietin-2, vascular endothelial growth factor A, fibroblast growth factor 2, fibroblast growth factor-21

## Abstract

*Background and Objectives*: Aortic valve stenosis (AS) develops with a pronounced local inflammatory response, where a variety of growth factors are involved in the process, and may have a pro-inflammatory and anti-inflammatory effect. The aim of our study was to elucidate whether circulating growth factors: growth differentiation factor 15 (GDF-15), angiopoietin-2 (Ang-2), vascular endothelial growth factor A (VEGF-A), fibroblast growth factor 2 (FGF-2), and fibroblast growth factor 21 (FGF-21) could be proposed as clinically relevant biomarkers to improve risk stratification in AS patients. *Materials and Methods*: AS patients were classified into three groups: 16 patients with mild AS stenosis; 19 with moderate and 11 with severe AS, and 30 subjects without AS (echocardiographically approved) were selected as a control group. GDF-15, Ang-2, VEGF-A, FGF-2, and FGF-21 were measured in plasma by the ELISA method. *Results*: GDF-15 levels differed significantly not only when comparing AS patients with control groups (*p* < 0.0001), but also a statistically significant difference was achieved when comparing AS patients at a mild degree stage with control individuals. We found a strong relationship of GDF-15 levels regarding AS severity degree (*p* < 0.0001). VEGF-A, FGF-2 and FGF-21 levels were significantly higher in AS patients than in controls, but relationships regarding the AS severity degree were weaker (*p* < 0.02). ROC analysis of the study growth factors showed that GDF-15 might serve as a specific and sensitive biomarker of AS stenosis (AUC = 0.75, *p* = 0.0002). FGF-21 correlated with GDF-15, Ang-2, and FGF-2, but it did not reach the level to serve as a clinically relevant biomarker of AS stenosis. *Conclusions:* AS is associated with significantly increased GDF-15, VEGF-A, FGF-2, and FGF-21 levels in plasma, but only GDF-15 shows a pronounced relationship regarding AS severity degree, and GDF-15 might serve as a specific and sensitive biomarker of AS stenosis.

## 1. Introduction

The prognostic utility of multiple biomarkers including cytokines and growth factors has been demonstrated in heart failure patients without aortic valve stenosis (AS). Since 2010, a number of new growth factors have been identified, with both diagnostic and prognostic relevance in AS patients. In these studies, there is practically no comparative study of growth factors in AS patients, particularly without other cardiovascular diseases.

There is evidence that serum growth differentiation factor 15 (GDF-15) is a clinically relevant inflammatory biomarker for several cardiovascular diseases, including hypertrophic cardiomyopathy [1,2,3]. Plasma levels of GDF-15 correlate with increased mortality after transcatheter aortic valve replacements [4].

Inflammation and angiogenesis are two closely related processes involving not only GDF-15 but also angiopoietin-2 (Ang-2), vascular endothelial growth factor (VEGF), fibroblast growth factor 2 (FGF-2) and other factors. GDF-15 is involved in angiogenesis and acute inflammation in an unstable atherosclerotic plaque [5]. Ang-2 is not only an important proangiogenic factor, but it is also involved in the development of inflammatory processes [6].

Pathological neovascularization is an important aspect of calcification of aortic valve disease, in which Ang-2 activates valvular endothelial cells (VECs) and valvular interstitial cells, promoting neovessel formation [7]. VEGF-A stimulates differentiation of myofibroblasts to osteoblasts in cusps of the aortic valve [8]. Data suggest that the serum levels of VEGF-A and Ang-2 can be directly correlated with each other [9].

FGF-2 plays a regenerative and an antifibrotic role in AS through increased matrix remodeling, proliferation, and the inhibition of profibrotic markers [10].

Anti-inflammatory growth factors such as FGF-21 (cardiomyokine) have also been identified. FGF-21 exerts cardioprotective effects, e.g., against cardiac hypertrophy and cardiac inflammation [11]. Data suggest that the blood levels of FGF-21 are associated with increased cardiovascular risk [12]. Our previous study found that circulating concentrations of FGF-21 have also been increased in AS patients without coronary and peripheral atherosclerosis [13].

It has been proven that AS develops with a pronounced local inflammatory response, including lesion of the VECs that leads to calcification [14]. We hypothesized that the manifestation of both inflammatory and anti-inflammatory growth factors is different at various stages of development of AS and may serve as clinical biomarkers. 

The aim of our study was to elucidate whether circulating growth factors—GDF-15, Ang-2, VEGF-A, FGF-2, and FGF-21—could be proposed as clinically relevant biomarkers to improve risk stratification in AS patients.

## 2. Materials and Methods

### 2.1. Study Subjects

Patients and controls were included according to the echocardiographically confirmed results, and the data were obtained using a GE VIVID 7 Dimension Cardiovascular Ultrasound system (GE Healthcare; GE Healthcare, Chicago, IL, USA) and Philips IE 33 Ultrasound Machine (Philips Healthcare, Amsterdam, The Netherlands). Each EchoCG examination was evaluated by two professionals. Patients with AS were subdivided into three groups depending on the severity grade according to current guidelines on the management of valvular heart disease and EchoCG criteria: aortic jet velocity (Vmax) (m/sec); mean pressure gradient, PG (mmHg); aortic valve area, AVA (cm^2^) and indexed AVA (cm^2^/m^2^). Data were graded as severe: Vmax > 4 m/sec, PG > 40 mmHg, AVA < 1.0 cm^2^, indexed AVA < 0.6; moderate: Vmax 3.0–4.0 m/sec, PG 20–40 mmHg, AVA 1.0–1.5 cm^2^, indexed AVA 0.60–0.85; and mild: Vmax 2.5–2.9 m/sec, PG < 20 mmHg, AVA > 1.5 cm^2^, indexed AVA > 0.85 [15]. 

Exclusion criteria for all the study participants were as follows: bicuspid aortic valve, pathologies of other valves, and rheumatic aortic valve disease (by EchoCG, history of rheumatism); cardiomyopathies, cardiac fibrosis, and left ventricular systolic dysfunction (EF below 50%); coronary atherosclerosis; peripheral atherosclerosis (by measuring intima–media thickness of carotid arteries, ankle–brachial index, history of peripheral artery disease and stroke); severe, moderate and uncontrolled arterial hypertension; diabetes mellitus, obesity, smoking; connective tissue diseases, infectious diseases, history of immune disease, oncological diseases; thyroid disfunction; and hypercholesterolemia and hypertriglyceridemia, including a history of statin and fibrate use.

AS patients were classified into three groups: 16 patients with mild AS; 19 with moderate AS; and 11 with severe AS, according to the 2012 European Society of Cardiology and the European Association for Cardio-Thoracic Surgery Guidelines for the Management of Valvular Heart Disease [16]. Thirty subjects without AS (echocardiographically approved) were selected as a control group. The study groups were matched by age and body mass index.

The clinical study was approved by the Riga Stradins University (Latvia) Ethics Committee on Research on Humans (No 12.09.2013/11). The study protocol conforms to the Ethical Guidelines of the 1975 Declaration of Helsinki, revised in 2008.

### 2.2. Laboratory Assays

Study subjects’ venous blood samples were collected after overnight fasting, centrifuged, and stored at −80 °C. GDF-15, Ang-2, VEGF-A, FGF-2, and FGF-21 were measured in plasma by the ELISA method using a TECAN Infinite 200 PRO multimode reader (Tecan Group, Ltd., Mannedorf, Switzerland). Concentrations of lipids, glucose, and other routine blood biomarkers were analyzed by standard methods.

### 2.3. Statistical Analysis 

Normal distributions of data were proved by D’Agostino and Pearson, Anderson–Darling, and Shapiro–Wilk normality tests. Homogeneity of variances was tested using F-tests (2 groups) or Brown–Forsythe and Bartlett’s tests. In most cases, dispersion was not corresponded as a normal distribution; therefore, the data were analyzed by a nonparametric Mann–Whitney (MW) U test or Kruskal–Wallis (KW) H test followed by the two-stage step-up method of Benjamini, Krieger, and Yekutieli as a post-hoc procedure, and results were displayed as the median and interquartile range (IQR). The data represented in Table 1 had a generally normal distribution; they were subjected to ANOVA parametric analysis with the corresponding post-test already mentioned. In a case of VEGF-A and FGF-2, the additional qualitative analysis of Fisher’s test of proportions was applied.

The correlation analysis was performed to determine the relationships between the studied growth factors and to clarify whether there were some associations between the severity of AS and the concentration of these factors. For better visualization of the direction of these associations, linear regression lines have been added on the corresponding scatter graphs. A *p*-value of less than 0.05 (*p* < 0.05) was considered statistically significant for all statistical tests.

The performance of the study biomarkers was assessed using receiver-operating characteristic (ROC) curves, sensitivity, specificity, and negative and positive predictive values. The *p*-value was reported for the area under the curve (AUC) for the best cut-off level. Diagnostic tests were assessed by this classification: 0.90–1 = excellent; 0.80–0.90 = good; 0.70–0.80 = fair; 0.60–0.70 = poor; and 0.50–0.60 = fail.

Statistical power calculations were performed with the GraphPad StatMate application. All graphical images and statistical analyses were performed using GraphPad Prism 9.0 for MacOS software (GraphPad Software, San Diego, CA, USA).

## 3. Results

### 3.1. Patient Characteristics

The basic data of the subjects included in the study are presented in Table 1. The average ages of patients in all aortic stenosis groups and in the control group were similar, and the mean body mass index (BMI) did not differ between the groups. The groups were similar regarding the mean values of the ejection fraction (EF) determined by the Simpson’s method and the stroke volume index (SVI) measured by the left ventricular outflow method, as well as according to the inclusion and exclusion criteria. 

### 3.2. GDF-15 Level Differences between the Patient Groups

**Figure 1:**
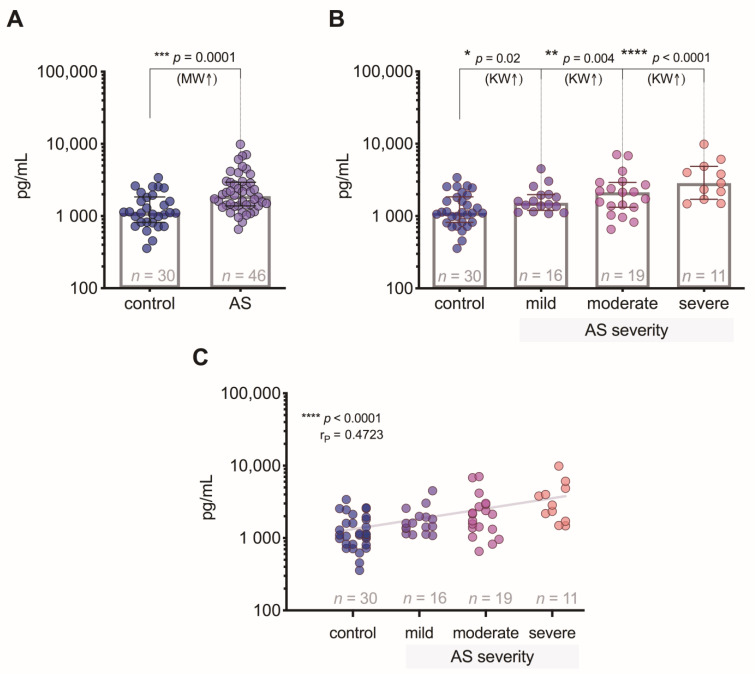
Plasma GDF-15 levels: (**A**) comparison of GDF-15 concentrations in control subjects and aortic valve stenosis (AS) patients; (**B**) comparison of GDF-15 concentrations in control subjects and AS patients with 3 different severity degrees; (**C**) correlation between plasma GDF-15 levels and the severity of AS (the gray line indicates a positive relationship). MW—Mann–Whitney U test; KW—Kruskal–Wallis test; r*_P_*—Pearson’s correlation coefficient.

Patients with AS had significantly higher levels (*p* = 0.0001) of GDF-15 compared to the controls (Figure 1A). GDF-15 levels were higher in the mild degree (*p* = 0.02), but the highest difference was reached between severe AS (*p* < 0.0001) compared to the controls (Figure 1B). The correlation analysis revealed a strong positive relationship (*p* < 0.0001) of GDF-15 levels regarding the degree of AS severity (Figure 1C).

### 3.3. VEGF-A Level Differences between the Patient Groups

**Figure 2:**
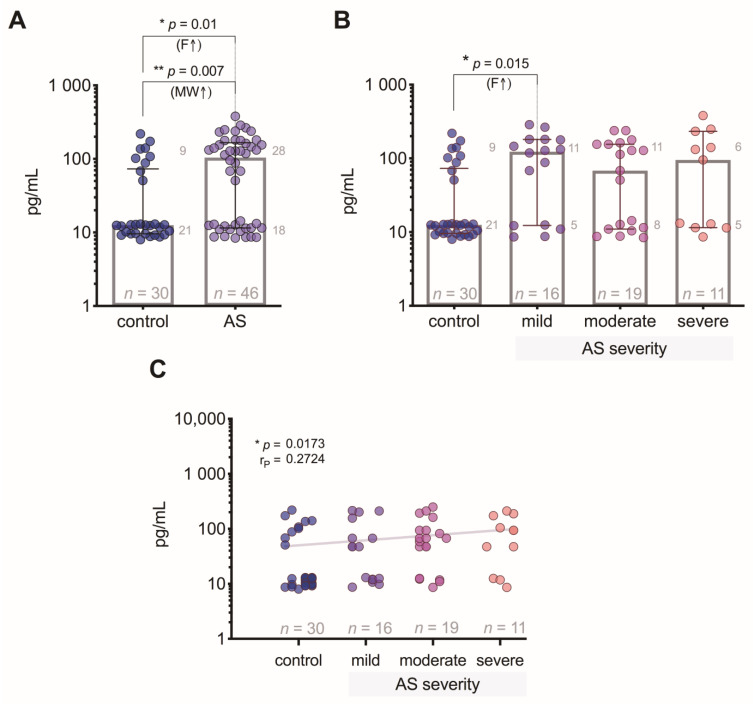
Plasma VEGF-A level: (**A**) comparison of VEGF-A concentrations in control subjects and aortic valve stenosis (AS) patients; (**B**) comparison of VEGF-A concentrations in control subjects and AS patients with 3 different severity degrees; (**C**) correlation between plasma VEGF-A levels and the severity of AS (the gray line indicates a positive relationship). MW—Mann–Whitney U test; F—Fisher’s exact test (small gray numbers show counts of cases with VEGF-A concentration ≤ 15 and >15 pg/mL); r*_P_*—Pearson’s correlation coefficient.

VEGF-A levels had a statistically significant difference (*p* = 0.01) between controls and AS patients (Figure 2A); however, regarding AS severity groups, a level of statistical significance (*p* = 0.015) was reached only between controls and patients with mild AS (Figure 2B). The correlation analysis showed a weak positive relationship (*p* = 0.017) of VEGF-A levels regarding the degree of AS severity (Figure 2C).

### 3.4. FGF-2 Level Differences between the Patient Groups

**Figure 3:**
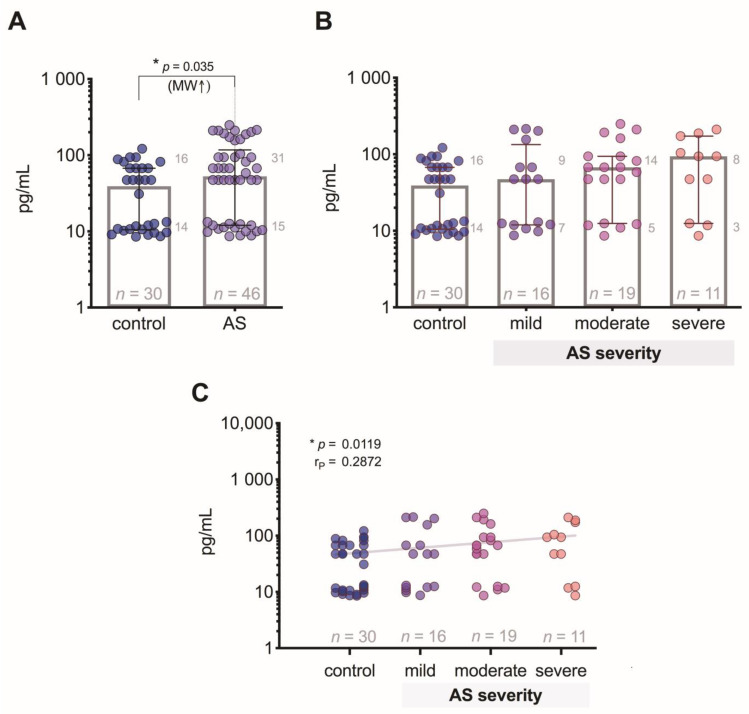
Plasma FGF-2 level: (**A**) comparison of FGF-2 concentrations in control subjects and aortic valve stenosis (AS) patients; (**B**) comparison of FGF-2 concentrations in control subjects and AS patients with 3 different severity degrees; (**C**) correlation between plasma PGF-2 levels and the severity of AS (the gray line indicates a positive relationship). MW—Mann–Whitney U test; r*_P_*—Pearson’s correlation coefficient.

We revealed that the FGF-2 level in AS patients compared to controls was increased (*p* = 0.035) (Figure 3A), but we did not find any statistically significant differences between controls and patient groups with different degrees of AS (Figure 3B), although the correlation analysis showed a weak positive relationship (*p* = 0.012) of FGF-2 levels regarding the AS severity degree (Figure 3C).

### 3.5. FGF-21 Level Differences between the Patient Groups

**Figure 4:**
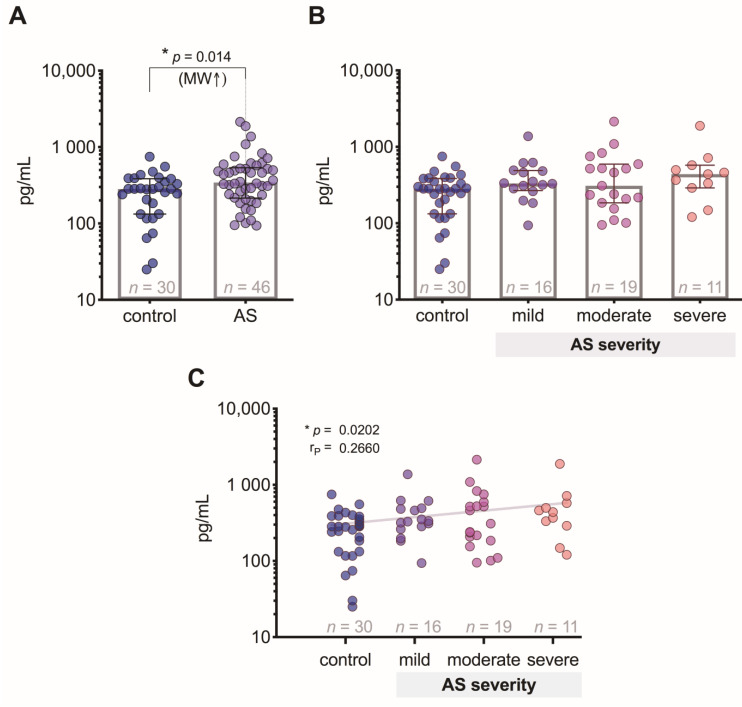
Plasma FGF-21 level: (**A**) comparison of FGF-21 concentrations in control subjects and aortic valve stenosis (AS) patients; (**B**) comparison of FGF-2 concentrations in control subjects and AS patients with 3 different severity degrees; (**C**) correlation between plasma PGF-21 levels and the severity of AS (the gray line indicates a positive relationship). MW—Mann–Whitney U test; r*_P_*—Pearson’s correlation coefficient.

FGF-21 levels were significantly (*p* = 0.014) increased in patients with AS compared to control subjects (Figure 4A), but we did not find any statistically significant differences between controls and patient groups with different degrees of AS (Figure 4B), although the correlation analysis showed a weak positive relationship (*p* = 0.02) of FGF-21 levels regarding AS severity degree (Figure 4C).

### 3.6. Ang-2 Level Differences between the Patient Groups

**Figure 5:**
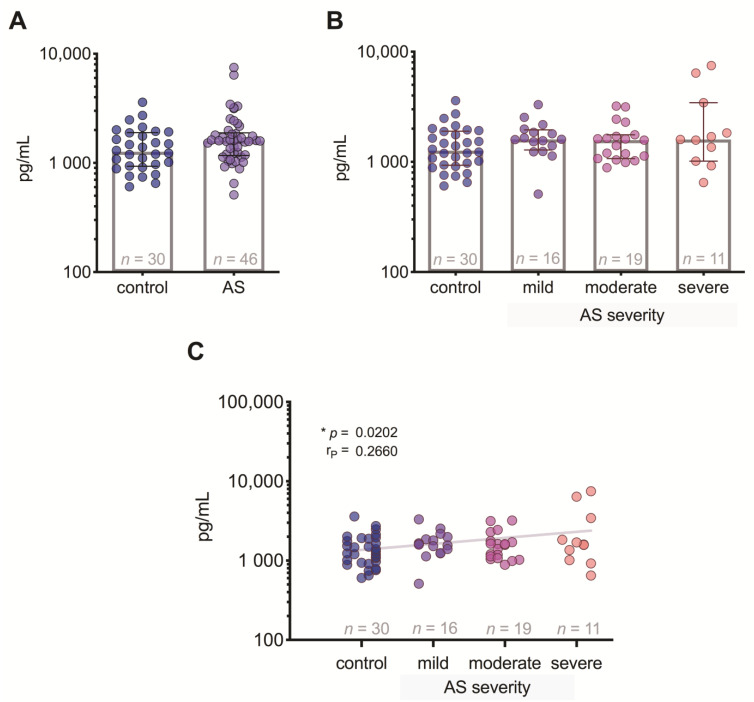
Plasma Ang-2 level: (**A**) comparison of Ang-2 concentrations in control subjects and aortic valve stenosis (AS) patients; (**B**) comparison of Ang-2 concentrations in control subjects and AS patients with 3 different severity degrees; (**C**) correlation between plasma Ang-2 levels and the severity of AS (the gray line indicates a positive relationship). r*_P_*—Pearson’s correlation coefficient.

In this study, we did not reveal significant differences between controls and AS patients (Figure 5A), or any statistically significant differences between the control group and patient groups at different stages of AS (Figure 5B), but the correlation analysis showed a weak positive relationship (*p* = 0.02) of Ang-2 levels regarding the degree of AS severity (Figure 5C).

### 3.7. Correlations between the Study Growth Factors

**Figure 6:**
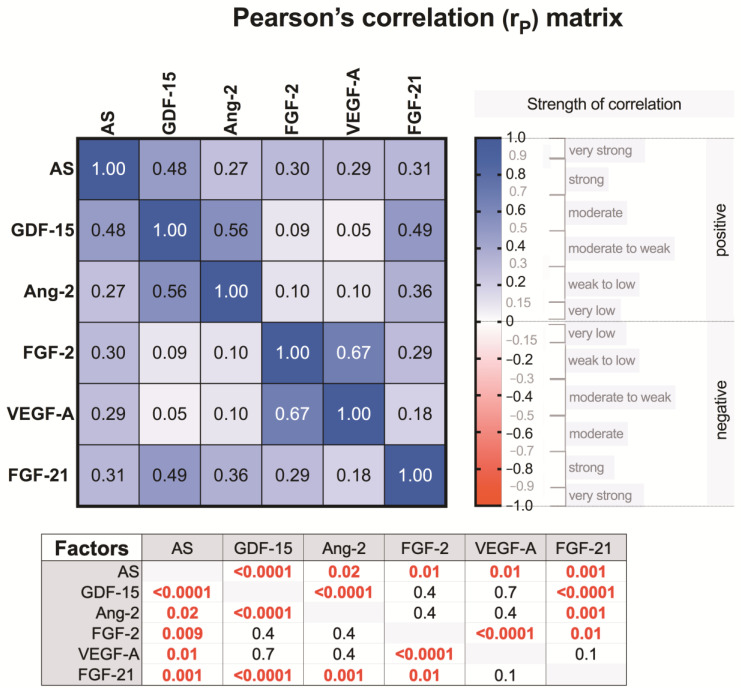
Correlation matrix representing the strength of the association between the levels of the growth factors and the severity (no, mild, moderate, severe) of AS (see upper first row or left first column), as well as covariances of the factors studied. The numbers in cells (squares) show the value of the Pearson’s correlation coefficient (r*_P_*); the table below represents the corresponding *p*-values.

In the correlation analysis between the growth factors in all study subjects (controls and AS patients), we found that FGF-21 was more correlated because it correlated with GDF-15 (*p* < 0.0001), Ang-2 (*p* < 0.001), and FGF-2 (*p* < 0.01). There were also the following correlations: GDF-15 correlated with Ang-2 (*p* < 0.0001), and FGF-2 correlated with VEGF-A (*p* < 0.0001) (Figure 6).

**Figure 7:**
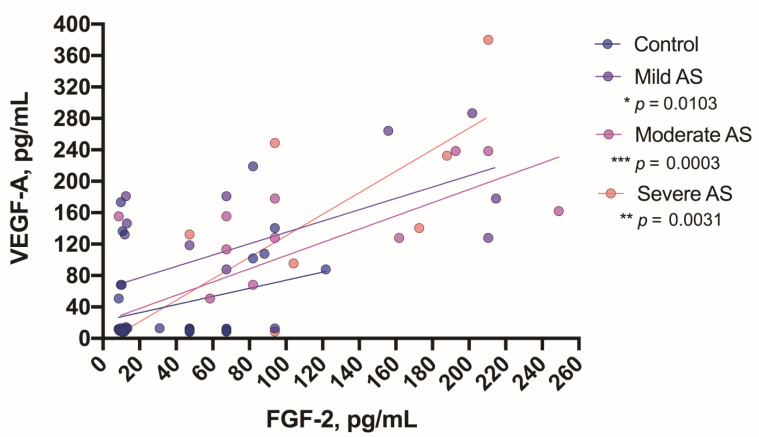
Correlation between FGF-2 and VEGF-A levels in the aortic valve stenosis patients. Asterisks show the level of statistical significance.

**Figure 8:**
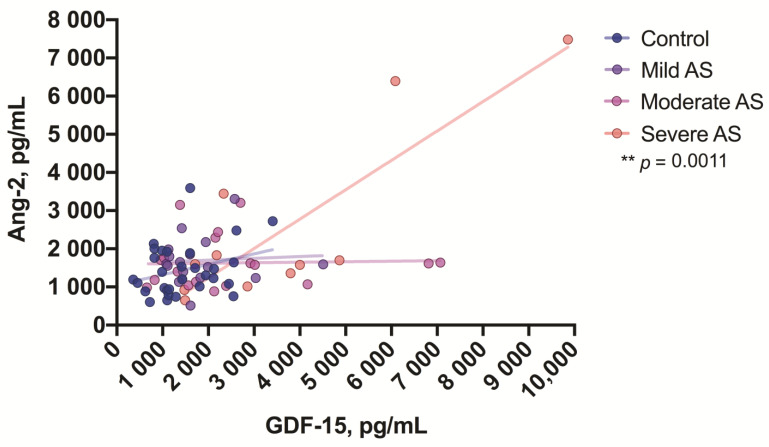
Correlation between GDF-15 and Ang-2 levels in the aortic valve stenosis patients. Asterisks show the level of statistical significance.

We also found some correlations between growth factors in the groups of mild, moderate, and severe AS patients. In all three patient groups, FGF-2 correlated with VEGF-A (mild *p* = 0.01, moderate *p* = 0.0003, and severe degrees of AS *p* = 0.003) (Figure 7), whereas in patients with a severe degree of AS, GDF-15 correlated with Ang-2 (*p* = 0.001) (Figure 8).

### 3.8. ROC Analysis of the Study Growth Factors

**Figure 9:**
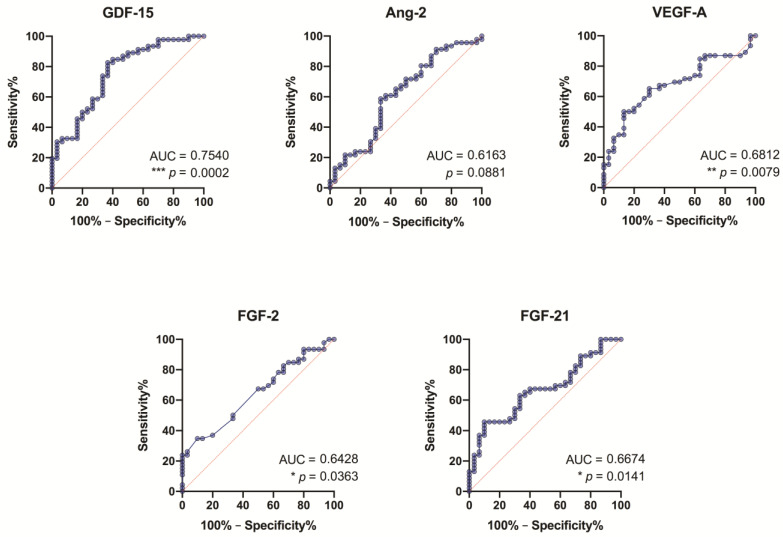
Receiver-operating characteristic (ROC) curves for growth differentiation factor 15 (GDF-15), angiopoietin-2 (Ang-2), vascular endothelial growth factor A (VEGF-A), fibroblast growth factor 2 (FGF-2) and fibroblast growth factor-21 (FGF-21) as diagnostic markers of aortic valve stenosis in the control subjects vs. all study patients. AUC—the area under the curve.

**Figure 10:**
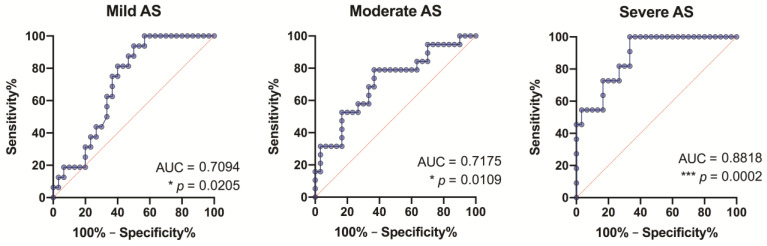
Receiver-operating characteristic (ROC) curves for growth differentiation factor 15 (GDF-15) as a diagnostic marker of aortic valve stenosis in the control group vs. all stenosis groups. AUC—the area under the curve.

Our findings (from the ROC analysis) suggest that all study growth factors, except Ang-2, might serve as specific and sensitive biomarkers for AS stenosis without grading the severity (Figure 9), although GDF-15 is more pronounced (fair level: AUC = 0.75, *p* = 0.0002). It should be noted that GDF-15 showed its significance in all degrees of AS, but most prominently in patients with severe AS (Figure 10). 

Analyzing the significance of other growth factors by AS degrees, we found that FGF-2 as a specific and sensitive biomarker reached a fair level only in severe degree AS patients (fair level: AUC = 0.71, *p* = 0.04); VEGF-A—in mild degree AS patients (fair level: AUC = 0.70, *p* = 0.02); and FGF-21—in severe degree AS patients (fair level: AUC = 0.75, *p* = 0.02).

## 4. Discussion

Although both GDF-15 and Ang-2 are proangiogenic and proinflammatory factors, their blood concentrations, correlation with other growth factors, and clinical significance differ in AS patients. Circulating GDF-15 levels differed significantly not only when comparing AS patients with control groups, but statistically significant differences were even achieved when comparing mild degree AS patients with control individuals. Although Ang-2 levels did not differ between AS patients and controls, the correlation analysis showed a weak positive relationship of Ang-2 levels regarding the degree of AS severity. It should be noted that Ang-2 correlated with GDF-15. ROC analysis of the study growth factors showed that GDF-15 might serve as a specific and sensitive biomarker for AS stenosis (fair level), and is especially seen in patients with severe AS, but Ang-2 is not significant. The clinical diagnostic significance of circulating GDF-15 in AS patients could be predicted from studies on its role in several cardiovascular diseases and mortality after transcatheter aortic valve replacement [1,2,3,4,17]. Stress conditions affect GDF-15 levels, and this, in turn, affects the regulation of myocyte hypertrophy and is therefore associated with the severity of hypertrophic cardiomyopathy [2]. GDF-15 serum levels have clinical utility in patients with left ventricular hypertrophy [1].

VEGF-A and FGF-2 are predominantly angiogenic markers where VEGF-A is significantly involved in calcific of aortic valve disease [8], while FGF-2 is involved in AS through increased matrix remodeling, proliferation, and inhibition of profibrotic markers [10]. For both markers, circulating levels were significantly higher in AS patients than in controls, but only VEGF-A was increased in patients with mild AS. Although there were no other differences in the severity of the AS degree for these two markers, VEGF-A and FGF-2 showed weak positive relationships regarding the degree of AS severity. We found that circulating levels of VEGF-A and FGF-2 directly correlate with each other, and FGF-2 also correlates with GDF-15 and Ang-2. Our results did not confirm the ability of these markers to serve as specific and sensitive biomarkers of AS stenosis. Both VEGF-A and FGF-2 could play a significant pathophysiological role in the development of AS, but without a significant increase in blood flow.

FGF-21 is an anti-inflammatory biomarker [11]. Our results suggest that FGF-21 levels are higher than in control individuals, and the correlation analysis showed a weak positive relationship of FGF-21 levels regarding AS severity degree (Figure 4). Interestingly, FGF-21 showed the highest number of correlations with the study growth factors—it correlated with GDF-15, Ang-2, and FGF-2 (Figure 6)—but it did not reach the level to serve as a specific and sensitive biomarker of AS stenosis (Figure 9). FGF-21 is also an endocrine factor that can be released into the circulation, and it affects angiogenesis associated with endothelial cells [18]. FGF-21 is produced by muscle fibers, and studies have shown that it improves skeletal muscle function [19], although FGF-21 is also produced by cardiomyocytes in a response to various stress factors [11]. Based on research data on the cardioprotective effect of this cardiomyokine, it can be hypothesized that FGF-21 may play a clinical role in inhibiting the pathogenesis of AS [11,12].

There were some limitations to the study, e.g., the study groups were small, but this was due to the use of a large number of exclusion criteria for the study participants with the aim of reducing effects on circulatory growth factors other than AS-related factors. Our results and correlations suggest that all five growth factors in the study are closely related, although their blood concentrations were differentially elevated, and they had different abilities to serve as specific and sensitive biomarkers of AS stenosis. The GDF-15 test can be used in patients with reduced LV ejection fractions and suspected severe aortic valve stenosis when echocardiography parameters correspond to moderate stenosis and no additional investigations have been performed, such as stress echocardiography. It could be used in all patients with mild aortic valve stenosis. The analysis should be performed once a year to predict the rate of stenosis progression, and possibly the time of surgical treatment.

## 5. Conclusions

AS is associated with significantly increased GDF-15, VEGF-A, FGF-2, and FGF-21 levels in plasma, but only GDF-15 show a pronounced relationship regarding the degree of AS severity, and GDF-15 might therefore serve as a specific and sensitive biomarker of AS stenosis.

## Figures and Tables

**Table 1:** Basic data of individuals in the control group and patients in the aortic valve stenosis (AS) groups.

		Controls *n* = 30	Mild Aortic Valve Stenosis *n* = 16	Moderate Aortic Valve Stenosis *n* = 19	Severe Aortic Valve Stenosis *n* = 11
Gender (%)	Male Female	6 (20) 24 (80)	1 (6) 15 (94)	8 (42) 11 (58)	7 (64) 4 (36)
Age (years)	Mdn (IQR)	70 (60–75)	72 (66–75)	74 (65–79)	69 (60–75)
^1^ BMI	M (±SD) *p*-value vs. control	27.97 (5.10)	29.53 (4.97) *p *= 0.16	27.18 (4.76) *p *= 0.19	27.02 (4.04) *p *= 0.30
^2^ SV (mL)	M (±SD) *p*-value vs. control	81.97 (22.20)	72.13 (11.99) *p *= 0.26	79.89 (20.45) *p *= 0.45	78.09 (17.39) *p *= 0.12
^3^ EF (%)	M (±SD) *p*-value vs. control	61.22 (6.44)	57.58 (9.79) *p *= 0.17	61.32 (8,24) *p *= 0.17	57.73 (8,65) *p *= 0.15
^4^ SVI	M (±SD) *p*-value vs. control	44.45 (11.19)	39.26 (8.39) *p *= 0.38	42.36 (11.67) *p *= 0.51	41.97 (9.99) *p *= 0.11

^1^ BMI—body mass index, weight in kilograms divided by the square of the height in meters (kg/m^2^); ^2^ SV—stroke volume, measured by left ventricular outflow method; ^3^ EF—ejection fraction, measured by Simpson’s method; ^4^ SVI—stroke volume index, the relationship between the stroke volume and size of the persons’ body surface area (mL/m^2^).

## Data Availability

The datasets used and/or analyzed during the present study are available from the corresponding author on reasonable request.

## References

[1] Hanatani S., Izumiya Y., Takashio S., Kojima S., Yamamuro M., Araki S., Rokutanda T., Tsujita K., Yamamoto E., Tanaka T. (2014). Growth differentiation factor 15 can distinguish between hypertrophic cardiomyopathy and hypertensive hearts. Heart Vessels.

[2] Montoro-García S., Hernández-Romero D., Jover E., García-Honrubia A., Vilchez J.A., Casas T., Martínez P., Climent V., Caballero L., Valdés M. (2012). Growth differentiation factor-15, a novel biomarker related with disease severity in patients with hypertrophic cardiomyopathy. Eur. J. Intern. Med..

[3] Toutouzas K., Stathogiannis K., Latsios G., Synetos A., Drakopoulou M., Penesopoulou V., Michelongona A., Tsiamis E., Tousoulis D. (2019). Biomarkers in Aortic Valve Stenosis and their Clinical Significance in Transcatheter Aortic Valve Implantation. Curr. Med. Chem..

[4] Kim J.B., Kobayashi Y., Moneghetti K.J., Brenner D.A., O’Malley R., Schnittger I., Wu J.C., Murtagh G., Beshiri A., Fischbein M. (2017). GDF-15 (Growth Differentiation Factor 15) Is Associated with Lack of Ventricular Recovery and Mortality after Transcatheter Aortic Valve Replacement. Circ. Cardiovasc. Interv..

[5] Wang J., Wei L., Yang X., Zhong J.J. (2019). Roles of Growth Differentiation Factor 15 in Atherosclerosis and Coronary Artery Disease. Am. Heart Assoc..

[6] Scholz A., Plate K.H., Reiss Y. (2015). Angiopoietin-2: A multifaceted cytokine that functions in both angiogenesis and inflammation. Ann. N. Y. Acad. Sci..

[7] Arevalos C.A., Berg J.M., Nguyen J.M., Godfrey E.L., Iriondo C., Grande-Allen K.J. (2016). Valve Interstitial Cells Act in a Pericyte Manner Promoting Angiogensis and Invasion by Valve Endothelial Cells. Ann. Biomed. Eng..

[8] Weiss R.M., Miller J.D., Heistad D.D. (2013). Fibrocalcific aortic valve disease: Opportunity to understand disease mechanisms using mouse models. Circ. Res..

[9] Steinvil A., Arbel Y., Topilsky Y., Barak L., Entin-Meer M., Levy R., Schwartz A.L., Keren G., Finkelstein A., Banai S. (2016). Sustained Elevation of Vascular Endothelial Growth Factor and Angiopoietin-2 Levels After Transcatheter Aortic Valve Replacement. Can. J. Cardiol..

[10] Rodriguez A.G., Schroeder M.E., Walker C.J., Anseth C.J. (2018). FGF-2 inhibits contractile properties of valvular interstitial cell myofibroblasts encapsulated in 3D MMP-degradable hydrogels. APL Bioeng..

[11] Planavila A., Redondo-Angulo I., Villarroya F. (2015). FGF21 and Cardiac Physiopathology. Front. Endocrinol..

[12] Lenart-Lipińska M., Duma D., Hałabiś M., Dziedzic M., Solski J. (2016). Fibroblast growth factor 21—A key player in cardiovascular disorders?. Horm. Mol. Biol. Clin. Investig..

[13] Lurins J., Lurina D., Tretjakovs P., Mackevics V., Lejnieks A., Rapisarda V., Baylon V. (2018). Increased serum chemerin level to predict early onset of aortic valve stenosis. Biomed. Rep..

[14] Soto M.E., Salas J.L., Vargas-Barron J., Marquez R., Rodriguez-Hernandez A., Bojalil-Parra R., Pérez-Torres I., Guarner-Lans V. (2017). Pre- and post-surgical evaluation of the inflammatory response in patients with aortic stenosis treated with different types of prosthesis. BMC Cardiovasc. Disord..

[15] Vahanian A., Iung B. (2012). The new ESC/EACTS guidelines on the management of valvular heart disease. Arch. Cardiovasc. Dis..

[16] Vahanian A., Alfieri O., Andreotti F., Antunes M.J., Barón-Esquivias G., Baumgartner H., Borger M.A., Carrel T.P., De Bonis M., Evangelista A. (2012). Guidelines on the management of valvular heart disease (version 2012): The Joint Task Force on the Management of Valvular Heart Disease of the European Society of Cardiology (ESC) and the European Association for Cardio-Thoracic Surgery (EACTS). Eur. J. Cardiothorac. Surg..

[17] Fabiani I., Santoni T., Angelillis M., Petricciuolo S., Colli A., Pellegrini G., Mazzei D., Pugliese M.R., Petronio A.S., De Caterina R. (2020). Growth Differentiation Factor 15 in Severe Aortic Valve Stenosis: Relationship with Left Ventricular Remodeling and Frailty. J. Clin. Med..

[18] Huang W., Shao M., Liu H., Chen J., Hu J., Zhu L., Liu F., Wang D., Zou Y., Xiong Y. (2019). Fibroblast growth factor 21 enhances angiogenesis and wound healing of human brain microvascular endothelial cells by activating PPARγ. J. Pharmacol. Sci..

[19] Raimondo D.D., Tuttolomondo A., Musiari G., Schimmenti C., D’Angelo A., Pinto A. (2016). Are the Myokines the Mediators of Physical Activity-Induced Health Benefits?. Curr. Pharm. Des..

